# Multimodal assessment of effects of urban environments on psychological wellbeing

**DOI:** 10.1016/j.heliyon.2023.e16433

**Published:** 2023-05-20

**Authors:** O. Baumann, B. Brooks-Cederqvist

**Affiliations:** Bond University, School of Psychology, Robina, Australia

**Keywords:** Built environment, Urban, Environmental psychology, Emotion, Wellbeing, Stress

## Abstract

The built environment is a ubiquitous factor of modern human life, which fundamentally affects human wellbeing. Most existing research on the psychological effects of urban environments is predominantly based on subjective self-report measures, which provide valuable insights into subjective experiences, but are also affected by conscious and subconscious bias. In the current study, we evaluate a multimodal approach to capturing wellbeing by employing objective mobile physiological and neurophysiological measurement technologies alongside self-reports to capture the impact of two different urban environments. Moreover, we endeavoured to comprehensively measure and, when possible, control the physical aspects of the environment. Our study specifically aimed to discover differences between psychological wellbeing indicators in adults across low-density vs. moderate-density urban environments. Data collection took place in two urban outdoor locations in Australia. Statistical comparison of the two locations indicated that low urban density led to comparatively higher levels of psychological wellbeing compared to moderate urban density. Self-reports indicated that the low-density environment led to higher levels of subjective feelings of comfort and safety and reduced levels of negative mood. In line with the subjective reports, in the low-density environment compared to the moderate-density environment, individuals showed higher levels of EEG theta activity, while EEG beta and heart rate measures were lower. The research outcome provides insights into how urban density affects people's wellbeing and showcases the benefits of employing ecologically-valid multimodal psychological-environmental measurement approaches to effectively evaluate the psychological impacts of built environments.

## 1 Introduction

The world's urban population is expected to increase by 70% by 2050, which will have significant impacts on the physical environment [[1], [2], [3]]. The ecological and economic benefits of denser urbanisation are undisputed, but there are also challenges for human psychological wellbeing, i.e. general mood, the presence of stress, and feelings of comfort and security [[4], [5], [6]]. However, so far, urban development planning has mainly considered functional and cost-effective factors [[7], [8], [9], [10]].

Urban density has been associated with an increased risk of health issues, such as sleep disturbance, asthma, and allergies [[11], [12], [13], [14], [15], [16], [17]]. Furthermore, studies have reported higher rates of anxiety, depression, and mood disorders in urban areas compared to rural areas [20]. More specifically, Knöll et al. [18] found that net building density (building coverage and height) was positively associated with urban stress, leading to feelings of insecurity.

Previous research, focused on comparing dense city environments with rural areas, found that more physical space and green areas tend to elicit fewer adverse effects regarding psychological wellbeing, thus fostering higher levels of overall quality of life as compared to dense urban areas [[19], [20], [21]]. In contrast, Berlyne's [22] psychological arousal theory suggests that environmental complexity stimulates pleasant feelings and sparks people's interest. Therefore, an optimal balance between simplicity and complexity needs to be present for an individual to gain hedonic value (i.e., enjoyment) from an environment; environments that are either too complex or too simple can promote stress and mental fatigue.

In keeping with theories that advocate for people's affinity for balanced stimuli, Kaplan's [19] attention restoration theory (ART) suggests that the sensory stimuli of natural green space decrease levels of stress, as individuals do not need to continuously process busy, irrelevant stimuli; hence, they feel more relaxed. Similarly, Ulrich et al.′s [21]. stress reduction theory (SRT) suggests that being in a natural environment increases positive emotions (e.g., interest and pleasure) while decreasing negative emotions (e.g., stress) by reducing physiological stress responses (e.g., elevated heart rate) and promoting wellbeing.

Prolonged stress can have adverse effects on individuals' psychological wellbeing and thus worsen many mental health problems [20,[23], [24], [25]]. Existing research on psychological effects of urban environments has mainly focused on comparing dense city environments with green areas [20,26,27] and predominantly includes subjective reports [28,29]. Therefore, there is a gap in the research and a lack of understanding of how variations in urban density impact people's psychological wellbeing, which the current study aims to address.

The specific question for the current project was whether there any significant differences between subjective (i.e., self-reports) and objective (i.e., heart rate and resting state EEG) indicators of psychological wellbeing in adults across urban environments with ‘low’ and ‘moderate’ building density.

We predicted that subjective indicators of psychological wellbeing would differ significantly across ‘moderate’- and ‘low’-density environments. Specifically, we predicted that self-reported psychological comfort, physiological comfort, and safety would be higher in a low-density environment as compared to a moderate-density environment. Additionally, it was predicted that participants would report higher positive mood and lower negative mood in the low-density environment as compared to the moderate-density environment. Moreover, we predicted that physiological indicators of psychological wellbeing would differ significantly across low- and moderate-density environments. Specifically, it was predicted that heart rate would be higher in the moderate-density environment as compared to the low-density environment. Regarding EEG activity, it was predicted that beta frequency power would be lower and theta frequency power would be higher in the low-density environment as compared to the moderate-density environment.

## 2 Method

### 2.1 Study design

The study utilised a within-subjects, counterbalanced AB/BA design over two locations: ‘low-density’ and ‘moderate-density’. Participants were arranged in a random sequence and allocated to start at either the ‘low-density’ or ‘moderate-density’ location to mitigate any confusion due to training or order effects [30]. The study included one independent variable: an urban built environment with two within-subject conditions (low building density and moderate building density). Three dependent variables were chosen for the study to obtain both subjective and objective measures of psychological wellbeing. The first was subjective comfort, operationalised by aggregated scores on psychological comfort, physiological comfort and safety questions from the self-administered six-item questionnaire. The second outcome variable was subjective mood (positive and negative affect), operationalised by scores on the Positive and Negative Affect Schedule (PANAS). The third dependent measure was physiological arousal as a marker of psychological wellbeing, operationalised by recorded heart rate data and global neural EEG (theta, alpha, beta bands) resting state data. As described above and in line with previous research, several environmental control variables were also recorded that could potentially affect wellbeing [31,32], i.e. humidity, temperature and sound pressure.

### 2.2 Study environment

**Fig. 1:**
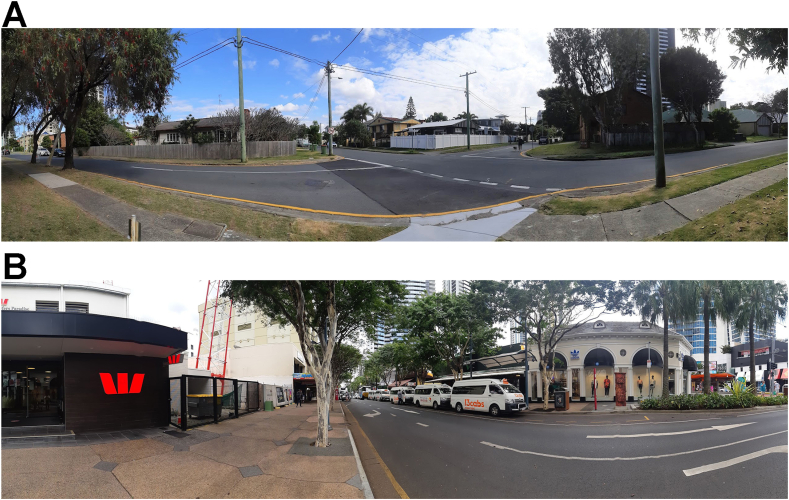
Environmental Locations. *Note*: Panoramic image of the (A) low building-density environment and (B) moderate-density environment.

The independent variable of urban built environment was operationalised in the form of the two Gold Coast city locations where data collection took place. According to the SpaceMatrix developed by Pont and Haupt [33] the two testing locations can be described as: low building-density and moderate building-density environments. The low-density area was a ‘less compact’ city area and the moderate-density environment was ‘more compact’. More specifically, the low-density environment was defined as having more open space (i.e., less urban land usage) and more native vegetation. The high-density environments were characterised as having higher urban land usage (i.e., less space and more high-rise buildings situated closer together) and exhibited less green areas; (Fig. 1A and B).

### 2.3 Environmental factors

Potentially confounding environmental variables, i.e., temperature & humidity (using a *TROTEC BP25* Pyrometer), and noise (using a *TROTEC BS06* sound level meter), were also measured to determine whether they significantly differed across the two locations (see Table 1; please also see the Procedure section for further details on our approach of controlling environmental variables).

**Table 1:** Means and standard deviations for environmental control measures.

	Low-density		Moderate-density	
	M	SD	M	SD
Temperature	23.65	2.06	23.44	2.31
Relative humidity	59.15%	9.27	61.76%	8.73
Average noise (LAeq)	47.68	2.46	58.91	1.83

*Note. M* = mean. SD = standard deviation. Temperature is expressed in Celsius (°C), and noise is expressed in A-weighted decibels (dBA).

A series of Shapiro-Wilk tests was performed on the environmental data and the assumption for normality was only satisfied (i.e. p > .05) for relative humidity. Therefore, to allow for greater consistency and comparability, the decision was made to use the more conservative, non-parametric Mann-Whitney *U* test for all measures [34]. Only acoustic noise was significantly different across locations (U = 0, p < .001). Importantly though, noise effects were already educed, if not eliminated, for the human observer by wearing noise-cancelling earplugs to reduce the differential effects of noise between locations (SLC80 26B; Australian standard; [35]). The earplugs provided noise reduction of 26 dB, reducing the noise of a busy street to that roughly equivalent to a quiet office (40 dB; [36]). Therefore, noise is not considered a variable of concern.

### 2.4 Participants

Participants were recruited via the Institution's Research Pool website. The sample size was estimated a priori using G*Power (version 3.1.9.6; [37]). To detect a medium effect size of d = 0.50, with the alpha level of significance set at 0.05 (two-tailed), and power at 80%, it was determined that 34 participants were required in a paired samples *t*-test [38,39]. The study's inclusion criteria stipulated that the participants be between 18 and 65 years of age, comprehend English fluently and have normal or corrected vision and hearing, based on self-reports. Eligible participants were required to be able to travel to and walk between the experiment's locations.

A total of 34 participants were recruited; however, due to hardware problems, EEG data were only available for 28 participants. Participants ranged in age between 19 and 63 years old (M = 27.20, SD = 11.40), 20 (59%) identified as female, 13 (38%) as male and 1 (3%) as other. Regarding educational background, 17 (50%) reported educational levels at year 12 or below and 17 (50%) reported educational levels at year 12 or above. Ethics approval was acquired via the Bond University Human Research Ethics Committee (Ethics approval number: #OB00032) and informed consent was obtained from all participants prior to the start of the experiment.

### 2.5 Psychological measures

#### 2.5.1 Self-reported comfort and safety

A custom-made questionnaire was used to measure the participants’ subjective evaluation of comfort and safety. The questionnaire consisted of six questions, utilising five-point Likert scales, regarding how comfortable participants felt in the moment: 1) Temperature: How do you feel dressed as you are? (Cold-Neutral-Hot); 2) Sun: Would you like more or less sunlight in the current environment? (Less-Neutral-More); 3) Humidity: How much humidity do you feel right now? (Low-Neutral-High); 4) Physical Comfort: How physically comfortable do you feel right now? (Not-Neutral-Very); 5) Psychological Comfort. How pleasant do you find the current environment? (Unpleasant-Neutral-Pleasant); 6) Safety: How safe do you feel here? (Unsafe-Neutral-Safe).

#### 2.5.2 The PANAS-SF

The 20-item International Positive and Negative Affect Schedule Short-Form (I-PANAS-SF [40]; was used to evaluate participants’ subjective experiences on two mood scales: positive affect (PA) and negative affect (NA). The instructions ask participants to indicate how they feel in the present moment on a five-point Likert scale spanning from ‘very slightly or not at all’ (1) to ‘extremely’ (5). The NA domain contains 10 items and requires participants to indicate to what degree they feel, for example, ‘upset’ or 'nervous'. Similarly, the PA section consists of 10 items, where they rate feeling ‘attentive’ or ‘inspired’. Lower NA scores represented lower levels of negative mood and higher scores represented higher levels of negative mood. Correspondingly, lower PA scores indicated lower levels of positive mood while higher scores were indicative of higher levels of positive mood.

The PANAS is one of the most widely used scales to measure mood or emotion, and has demonstrated sound psychometric properties (α = 0.85-0.87; [40,41]. Moreover, it also demonstrated acceptable model fit indices (CFI) on affect measures (CFI = 0.97, p < .001 [42]; CFI = 0.92, p < .001 [43]). Additionally, Crawford and Henry [44] showed convergent and discriminant validity (n = 1,003) with the depression, anxiety and stress scale (DASS; [45]. Results indicate significant positive correlations between the DASS and the NA scale (depression r = .60, anxiety r = 0.60), while showing moderate negative correlations between the DASS and the PA scale (depression r = −0.48, anxiety r = −0.30). Von Humboldt et al. [46] demonstrated external validity with a non-significant difference (χ2 2 (18) = 18.32; p = .435) in two independent groups with an older adult population (n = 1291). Melvin and Molloy [47] found moderately good internal consistency for the NA scale (a = 0.87) and PA scale (a = 0.87), which is similar to Watson et al.‘s [40], ranging from a = 0.84–0.87 for NA and a = 0.86–0.90 for PA. Lastly, Serafini et al. [43] assessed test/retest reliability and found high correlation coefficients when comparing baseline PANAS scores to post-treatment scores for NA (r = 0.67) and PA (r = 0.80).

#### 2.5.3 Heart rate measurement

Heart rate was measured as BPM using a mobile Elite HRV CorSense device, then further analysed using the Elite HRV software. The normal resting heart rate for young to older adults is between 60 and 100 BPM [48]. Previous studies have indicated that a temporarily elevated heart rate can be an objective arousal/stress indicator [48,49]. Several existing studies have employed mobile heart rate devices, as they are easy to use, non-invasive, inexpensive and sufficiently accurate [[50], [51], [52]]. The Elite HRV software employs its own proprietary signal quality algorithms, which detect, log and correct artifacts (i.e., false heartbeats; [[51], [52], [53]].

#### 2.5.4 EEG measurement

The frequency of EEG signals usually ranges from 0.01 Hz to around 100 Hz, which can be divided into five frequency bands: gamma (>30 Hz), beta (13–30 Hz), alpha (8–12 Hz), theta (4–7 Hz) and delta (<4 Hz; [54]). This study focused on midrange EEG frequencies (theta, alpha, beta), since these are typically considered more reliable [55]. Brain potentials were recorded using the Muse EEG portable headband device.

The devices had four main dry sensor electrodes: TP9 (left temporal-parietal) and TP10 (right temporal-parietal), which recorded spontaneous electrical brain activity on the outer side of the forehead, and AF7 (left anterior frontal electrode) and AF8 (right anterior frontal electrode), which recorded the frontal areas of the forehead [56]. Previous studies have shown that modern portable Muse EEG devices can reliably replicate EEG effects related to emotion and stress in outdoor settings [56,57]. Data were recorded using Mind Monitor Version 2.2 software at a sampling frequency of 256 Hz. A 50 Hz notch filter was implemented to filter out power line noise. Recorded files were saved in proprietary Muse raw data format and analysed with a custom-made Python program (Mobile EEG analysis; MEEGAN). The EEG channels were bandpass-filtered into the four frequency bands for data processing and visual inspection using an FIR filter applied forward and backward for zero-phase shift. To quantify signal power, we calculated the power spectral density (PSD) across all frequencies of interest using Welch's method with a 1-s window size [58], then determined the approximate average power within each frequency band by integrating the relevant frequencies using Simpson's rule.

### 2.6 Procedure

As described above, the independent variable of the environment was operationalised in the form of the two Gold Coast city locations where data collection took place. The ‘low-density’ area was a less compact city area, and centrally located in a residential community on Oak Avenue. The ‘moderate-density’ environment was more compact and situated on Cavill Avenue closer to the city shopping centre. Both areas were close to each other (approximately 20-min walking distance).

Participants were provided with a starting location in a counterbalanced sequence. Upon arrival, participants were given an explanatory statement outlining the study and their participation rights and were then required to provide written consent to continue the study. Participants were advised that participation was voluntary. Additionally, they were asked to fill out a questionnaire regarding demographic information.

To ensure standardisation, operational procedures remained the same for all participants aside from the order of location. Potentially confounding environmental variables (i.e., temperature, humidity, and noise) were also measured to determine whether they significantly differed across the two locations, thus affecting results ([59]; Table 1). The chair was set in a shaded location to avoid any effects of sunlight and participants were provided foam-tip, disposable, noise-cancelling earplugs to reduce the differential effects of noise between locations (SLC80 26B; Australian standard; [35]). The earplugs provided noise reduction of 26 dB, reducing the noise of a busy street to that roughly equivalent to a quiet office (40 dB; [36]). Therefore, noise is not considered a variable of concern.

At the beginning of each trial, participants were asked to complete the I-PANAS-SF to assess mood and the comfort and safety questionnaire. The participants were then guided around the area for 1 min, then seated comfortably in a portable chair, where they visually inspected the environment for 5 min. Participants were instructed on how to use the EEG headband and the CorSense heart rate measuring device. Once all physiological instruments were in place, participants were asked if they had any additional questions. Following this, they were asked to stay seated in a relaxed comfortable position with their eye lids closed for 10 min, during which EEG and heart rate were recorded. Closed eyelids ensured that EEG signals were not confounded by any sudden visual stimuli (e.g., passing people). During this phase, one researcher monitored the participants while a second logged onsite environmental factors (i.e., temperature, humidity and noise). After the procedure, the participants were debriefed and walked to the second location to undergo the same sequence. The experiment required approximately 15 min per location for data collection (approximately 45 min in total including travel between the two locations).

### 2.7 Statistical analysis

Statistical analyses were conducted with the IBM SPSS-27 program, while Bayesian data analyses were conducted with Jamovi-1.6. The study used an alpha level of 0.05 to assess significance. The main statistical analysis evaluated subjective and objective responses on each level of the independent variable (i.e., ‘low-density’ and ‘moderate-density’ environments).

#### 2.7.1 Data screening

Missing data analyses indicated a loss of five participants' EEG measures for matched pairs due to poor electrode contact. The presence of univariate outliers was assessed using SPSS box plots. For all variables of the data set, only one extreme outlier was identified in the alpha frequency band (i.e., 3 × interquartile range (IQR)). The data were retained, however, as the value were judged to be genuine (i.e., not affected by measurement error or artifacts).

#### 2.7.2 Normality

A series of Shapiro-Wilk tests was performed and the assumption for normality was only satisfied (i.e. *p* < .05) for self-reports of positive affect and EEG alpha frequency data. Therefore, to allow for greater consistency and comparability, the decision was made to use the more conservative, non-parametric Wilcoxon test for all measures [34].

## 3 Results

### 3.1 Results for subjective measures

#### 3.1.1 Comfort and safety questionnaire

Three paired Wilcoxon t-tests were used to assess differences in mean scores on comfort and safety between environments (*n* = 34). The results show that participants reported statistically significant higher scores on psychological comfort in the ‘low-density’ environment (*M* = 4.26, *SD* = 0.79) compared to the ‘moderate-density’ environment (*M* = 3.00, *SD* = 1.23, *Z* = 3.81, *p* < .001). Safety evaluations revealed that participants felt significantly safer in the ‘low-density’ location (*M* = 4.53, *SD* = 0.71) compared to the ‘moderate-density’ location (*M* = 3.44, *SD* = 1.08, *Z* = 4.13, *p* < .001). Lastly, in the ‘low-density’ environment, participants reported feeling statistically significantly more physical comfort (*M* = 4.12, *SD* = 0.95), as compared to the ‘moderate-density’ environment (*M* = 3.50, *SD* = 1.02, Z = 2.71, *p* = .007).

#### 3.1.2 Positive affect and negative affect - PANAS

Two paired Wilcoxon t-tests were used to contrast differences in mood (n = 34), while a Bayesian paired samples *t*-test was used to support non-significant results. Results for positive affect indicate that there was no significant difference between the ‘low-density’ environment (*M* = 30.76, *SD* = 7.41) and the ‘moderate-density’ environment (*M* = 30.15, *SD* = 8.08, *Z* = 1.02, p = .314). A Bayesian paired samples *t*-test was conducted for accuracy, which showed an odds ratio of BF01 = 6.21:1, demonstrating an 86.13% probability in favour of the null model being correct as compared to the alternative model. For negative affect, however, the results indicate that participants experienced decreased negative mood (*M* = 14.24, *SD* = 5.37) in the ‘low-density’ environment as compared to the ‘moderate-density’ environment (*M* = 15.53, *SD* = 5.11, Z = 1.99, *p* = .047).

## 4 Results for objective measures

### 4.1 Heart rate

Two paired Wilcoxon tests demonstrated that participants' (*n* = 34) heart rate responses were significantly lower (*M* = 72.0, *SD* = 9.17) in the ‘low-density’ environment compared to the ‘moderate-density’ environment (*M* = 74.65, *SD* = 10.85, Z = 2.36, *p* = .017).

### 4.2 EEG

Paired Wilcoxon tests (*n* = 29) were conducted to evaluate differences between average amplitude measures. Non-significant results were further assessed with Bayesian statistics. The study found that beta activity was statistically significantly lower in the ‘low-density’ environment (*M* = 0.11 *SD* = 0.02) as compared to the ‘moderate-density’ environment (*M* = 0.12 *SD* = 0.03, Z = 2.91, *p* = .003). Theta waves, however, were statistically significantly higher in the ‘low-density’ environment (*M* = 0.51 *SD* = 0.07) as compared to the ‘moderate-density’ environment (*M* = 0.48 *SD* = 0.11, Z = 2.14, *p* = .031). Lastly, there was no significant difference between scores on alpha activity between the ‘low-density’ environment (*M* = 0.12, *SD* = 0.03) and ‘moderate-density’ environment (*M* = 0.12, *SD* = 0.04, *Z* = 0.35, *p* = .736). A Bayesian paired samples *t*-test showed an odds ratio of BF01 = 6.97:1, which demonstrates an 87.45% probability of the null model being correct.

## 5 Discussion

The present study investigated psychological wellbeing across urban environments by utilising subjective and objective measurement tools. More specifically, it was hypothesised that both subjective indicators and physiological indicators of psychological wellbeing would significantly differ across ‘moderate-density’ and ‘low-density’ environments. The results of this study partly support the hypotheses.

As predicted, participants reported higher levels of psychological and physiological comfort as well as safety in the ‘low-density’ environment, as measured by the comfort and safety questionnaire. Existing research does not always clarify whether potential confounding environmental variables were assessed [20,29,60]. Therefore, to ensure the robustness of the current study, temperature, humidity, and noise were either measured or experimentally controlled for [59]. Taken together, the current study results indicate that participants' self-reported experience was affected by the density of the built environment rather than environmental factors.

Our findings are in line with previous research, which also reported associations of higher density of buildings with perceived urban stress [18]. One explanation is that building density tends to decrease open space in inner cities, which hinders traffic flow and increases areas of high vehicle congestion, which pedestrians perceive as unsafe [3,61]. This suggests that perceiving an environment as unsafe could lead to increased subjective stress and psychological discomfort.

Secondly, participants' heart rates were found to be significantly higher in the ‘moderate-density’ as compared to the ‘low-density’ environment. These results suggest that individuals were in a less aroused, more relaxed state within the ‘low-density’ condition while experiencing higher levels of arousal in the ‘moderate-density’ condition. This is in line with research finding that psychological states (e.g., feeling unsafe) that elicit short-term stress response and arousal manifest themselves in physiological changes (e.g., elevated heart rate [[62], [63], [64]]. The finding of increased heart rate in the ‘moderate-density’ condition by itself only indicates that individuals showed higher physiological arousal, not whether this arousal was associated with a positively or negatively valanced emotional state stressor (i.e., excitement or distress, respectively). However, when considering the self-report results, where participants experienced higher levels of security and psychological comfort as well as physiological comfort in the ‘low-density’ environment, it is indicated that the higher heart rate in the ‘moderate-density’ environment is caused by higher levels of distress rather than positive emotions (i.e., excitement).

Further, as hypothesised, participants displayed lower levels of beta frequency power and higher levels of theta frequency power in the ‘low-density’ as compared to the ‘moderate-density’ environment. In reference to earlier literature, these results indicate that individuals exhibited less physiological arousal and more relaxation in the ‘low-density’ condition [62,65,66]. The predominant theta activity in the ‘low-density’ environment suggests that participants were in an awake, relaxed state (or even daydreaming), while the predominant beta activity in the ‘moderate-density’ environment implies that participants had to increase their cognitive demand due to increased thinking and information processing. Increased beta activity is also suggestive of increased vigilance [66]. Considering that participants reported feeling less secure in the ‘moderate-density’ environment, beta activity may have increased as a function of vigilance and increased demand for attention [62]. Further support is provided by Berlyne's psychological arousal theory, which states that an optimal balance between complexity and simplicity induces pleasant feelings [22]. Here, findings show that individuals had less cognitive demand due to decreased information processing and, thus, arousal in the ‘low density’ environment. In turn, this suggests that the ‘low-density’ environment was closer to an optimal balance between complex and simple stimuli as compared to the ‘moderate-density’ condition. Finally, although this was not a dedicated hypothesis of this paper, as predicted, findings from also showed that participants displayed similar levels of alpha frequency power across both environments, demonstrating that the predominant factor explaining alpha activity was that participants had their eyelids closed in both conditions [66,67].

In regard to self-rated mood (as assessed by the PANAS scale), participants reported significantly lower levels of negative affect in the ‘low density’ environment but indicated no meaningful difference in levels of positive affect across both environments. While these findings only partially support our prediction, it is still a notable finding, as individuals' levels of positive mood did not appear to be influenced by the environment. This could indicate that the short duration of presence at the location might not have been enough to affect positive mood or that the ‘low-density’ environment lacked some qualities that could lead to such an increase. Nevertheless, participants' levels of negative mood were affected by urban density, showing lower levels of negative mood in less dense environments. This finding is supported by Berlyne's [22] psychological arousal theory, which stipulates that stimulation within an environment is good, yet too much or too little of it can be harmful by causing increased levels of stress and mental fatigue. Here, the ‘low-density’ environment may have been the right balance between stimuli, while the ‘moderate-density’ environment may have been less balanced, thus inducing higher levels of negative mood. Emotional arousal and degree of attention activation are also important in the association between stimulus and cognitive response, as these control the selection, organisation, and execution of a stress response [68] 2009).

Further, considering literature from Lazarus and Folkman [69], which specifies that psychological distress is dependent on the subjective interpretation of a perceived environment, whether it elicits positive or negative emotions depending on how demanding the experience is, it becomes clear that the balance between too much and too little stimulation and complexity is a subjective experience that can vary with people's preferences. Overall, these findings show a general preference towards low-density environments in the sample of participants while still indicating that moderate-density environments have a more adverse influence on psychological wellbeing.

The main strength of our study is that it does not rely on subjective or objective measures alone. Previous research has mostly used subjective assessments [29], which pose a challenge when analysing results systematically, as they commonly involve cognitive biases despite acceptable psychometric validation of instruments [70]. However, by including objective measurements, the study was able to identify and assess valuable relationships between low-density and moderate-density urban environments and psychological wellbeing more comprehensively. This mixed-method design improved the validity and reliability of the findings and strengthened the ability to discuss causal inferences [30]. For example, the measure of heart rate as the criterion for psychological wellbeing validates the self-reported measures on the comfort and safety questionnaire, as it addresses to what extent scores on the subjective scale agree with the objective measures. Moreover, we did our best to measure, balance, control and eliminate external factors such as temperature, humidity, and noise. It should be noted, though, that the complexity of real-life environments will never provide the high levels of experimental control and internal validity as simulations or laboratory environments. In addition, while the choice to eliminate, or at least reduce, the effects of noise controlled an important confound, it also reduced the ecological validity of the investigation. Similarly, our choice to collect EEG and data while participants had their eyes closed allowed us to measure the lasting emotional impact of the environments without the threat that visual occurrences could contaminate the EEG signals. However, the absence of visual information could have reduced the size of the emotional effect.

Another limitation of our study is that we assessed low to moderate density environments only. It will be necessary also to explore medium to high-density urban environments to determine the potential differences in stress impact. A more comprehensive assessment of different levels of urban density would also allow determining whether its relationship between psychological wellbeing is linear or nonlinear. Finally, our findings might be culturally specific, i.e. only apply to the Australian context. Therefore, capturing socioeconomic population characteristics may be needed to further extend this study's relevance to a wider range of contexts.

The current study has increased the diversity of the current body of research and contributed to a better understanding of how different urban environments impact psychological wellbeing. Even so, further research in this area, coupled with the current pace of newly developed mobile technology, will lead to even more all-encompassing, data-driven technological advancements suitable for recording brain signals or other physiological markers of stress and wellbeing in the field. More specifically, portable devices that capture multimodal data may hold the potential to better identify mental health risk factors and produce reliable data to improve individual health and public wellbeing. As a result, city planners and authorities can use these enhanced technologies in large-scale, real-time studies to create smart cities that sustain and promote human health. This will have great implications, as populations are growing, urban density is expanding, and green environments will ultimately become a luxury.

## 6 Conclusions

To conclude, we still do not know exactly how future urban growth will present in terms of spatial development in different parts of the world or its impact on human wellbeing and the environment. The objective of the current study was to identify any differences between urban environments as opposed to green vs. urban environments. The current study found meaningful differences between low- and moderate-density environments, using both subjective and objective psychological wellbeing indicators. As such, this paper contributes to new empirical insights and perspectives concerning how compact city environments impact individuals' wellbeing. It is important to point out that the current research results show that moderate-density environments are not necessarily unfavourable. In fact, we may speculate that they may even be preferred if they provide an optimal level of stimulation, which can enhance individuals' feelings of happiness, increase psychological wellbeing, and ultimately increase life satisfaction. Additionally, the experienced atmosphere of built environments depends on qualitative and quantitative elements. Taken together, the current study's findings suggest positive outcomes of urban density on psychological wellbeing. However, researchers and city developers should proceed with caution and consider how cities can optimise sustainability and human health and wellbeing. Individuals perceive lower degrees of urban density as less stressful. Moreover, our study shows that mobile physiological measurement technology provides reliable and ecologically valid indicators of psychological wellbeing. Our study also highlights that triangulation via objective and subjective measures provides broader information base and greater confidence for decision making.

## Declarations

### Ethics approval

All procedures performed in this study were approved by Bond University Human Research Ethics Committee (Ethics approval number: #OB00032) and are accordance with the ethical standards as laid down in the 1964 Declaration of Helsinki and its later amendments.

### Author contribution statement

Oliver Baumann: Conceived and designed the experiments; Analyzed and interpreted the data; Wrote the paper.

Briana Brooks-Cederqvist: Analyzed and interpreted the data; Wrote the paper.

### Data availability statement

Data associated with this study has been deposited at Open Science Framework (https://osf.io/bmd2w/?view_only=b0e4bcfdf17d4b09aa10d64a2ab8c085).

## Declaration of competing interest

The authors declare that they have no known competing financial interests or personal relationships that could have appeared to influence the work reported in this paper.
